# Improving the mechanical properties of a high density carbon block from mesocarbon microbeads according to oxidative stabilization

**DOI:** 10.1038/s41598-018-26971-8

**Published:** 2018-07-23

**Authors:** Ui-Su Im, Jiyoung Kim, Byung-Rok Lee, Dong-Hyun Peck, Doo-Hwan Jung

**Affiliations:** 1 Advanced Energy and System Engineering, Korea University of Science and Technology, Yuseong-gu Daejeon, 305350 Republic of Korea; 2 School of Chemical Engineering, Sungkyunkwan University, Jangan-gu, Suwon-si, Gyeonggi-do 440746 Republic of Korea; 3 New & Renewable Energy Research Division, Korea Institute of Energy Research, Yuseong-gu Daejeon, 305350 Republic of Korea

## Abstract

In this study, a high density carbon block without binder was manufactured by mesocarbon microbeads (MCMB) from coal tar pitch. To develop the high density carbon block without a binder, MCMBs were oxidized at different levels of temperature. To verify the effect of oxygen content in the carbonized carbon block (CCB), an elementary analysis (EA) and X-ray photoelectron spectroscopy (XPS) were performed. The morphological and mechanical properties of the CCBs were investigated by using scanning electron microscopy (SEM), a shore hardness test, and a flexural strength evaluation. The results revealed that the oxygen content increased with stabilization temperature and the physical properties of the CCBs were considerably improved via oxidative stabilization. Small cracks between MCMB particles were observed in the CCBs that were stabilized over 250 °C. From the results of this study, the CCB from MCMBs stabilized at 200 °C for 1 h showed optimum mechanical properties and high density.

## Introduction

High-density and high-strength isotropic carbon blocks are materials that are utilized in modern high-tech industries due to their low electrical resistance and excellent mechanical properties at high temperatures^1^. In particular, high density and high strength isotropic carbon blocks are used as adequate materials in nuclear reactors, electrical contacts, electrodes, refractories, crucibles for chemicals, and semiconductors^2–4^.

High density isotropic carbon blocks have been produced by several methods. One method is to use fillers such as coke and binder pitch. This method entails repetition of calcination and impregnation, which leads to long manufacturing time. To reduce the manufacturing time, self-sinterable coke or mesophase pitch has been used. These raw materials are formed and carbonized via a cold isostatic pressing method without a binder in order to shorten the process time^5–8^. Also, MCMBs have been utilized as raw materials by isostatic pressing and carbonization. These alternative methods reduced the processing cost and made the workforce efficient. Among three alternatives, the method using MCMBs is especially easy to produce high density due to the spherical shape of the MCMBs. In particular, since 1973, the MCMBs has been frequently used by many researchers as precursors of high-density high-strength carbon blocks and rechargeable Li-ion batteries^9–17^.

The effect of oxidation stabilization on mesophase pitch has been reported in several studies^18–21^. The reported mechanism of the oxidative stabilization is explained in three steps. Initially, methylene hydrogens are reduced. Aldehyde ketone and carboxylic acid functionality are subsequently produced. Finally, ester and anhydride functionality are increased^18^. These oxidation mechanisms are used for different purposes in different application fields such as MCMBs, carbon fibers, isotropic pitch, and graphene.

Oxidative stabilization in the field of carbon fiber increases the carbonization yield and yields excellent mechanical properties^19,22^. When producing isotropic pitch having high aromatic carbon content, air blowing is applied by exploiting an oxidation mechanism^18,20,23,24^. The oxidation process is also used in the graphene process developed by Hummers^25–27^. The oxidative process was implemented for effective exfoliation of graphene oxide in Hummers method, and this approach has the disadvantage restacking occurs easily during reduction at about 700–1000 °C due to a π-π interaction^28–30^. Also, the effect of oxidative stabilization on sintering of MCMBs during carbonization was studied in according with light and strong oxidative conditions. The oxidative stabilization in MCMB resulting in the transgranular and intergranular cracks was investigated^8^.

In the present study, oxygen content produced via oxidative stabilization was used as an additive to facilitate carbon-carbon bonding so that high-density and high-strength isotropic carbon blocks without a binder were developed from MCMBs prepared from coal tar pitch. The correlation between the carbon block shrinkage, the time of crack formation, and the weight loss ratio according to the temperature range were investigated from the difference of added oxygen ratio. The swelling phenomenon was inhibited by increasing the aromatic carbon content and the amount of volatile matter was controlled. Optimum conditions to improve the mechanical properties were finally studied.

## Results and Discussion

### Preparation of MCMBs with a uniform size

**Figure 1:**
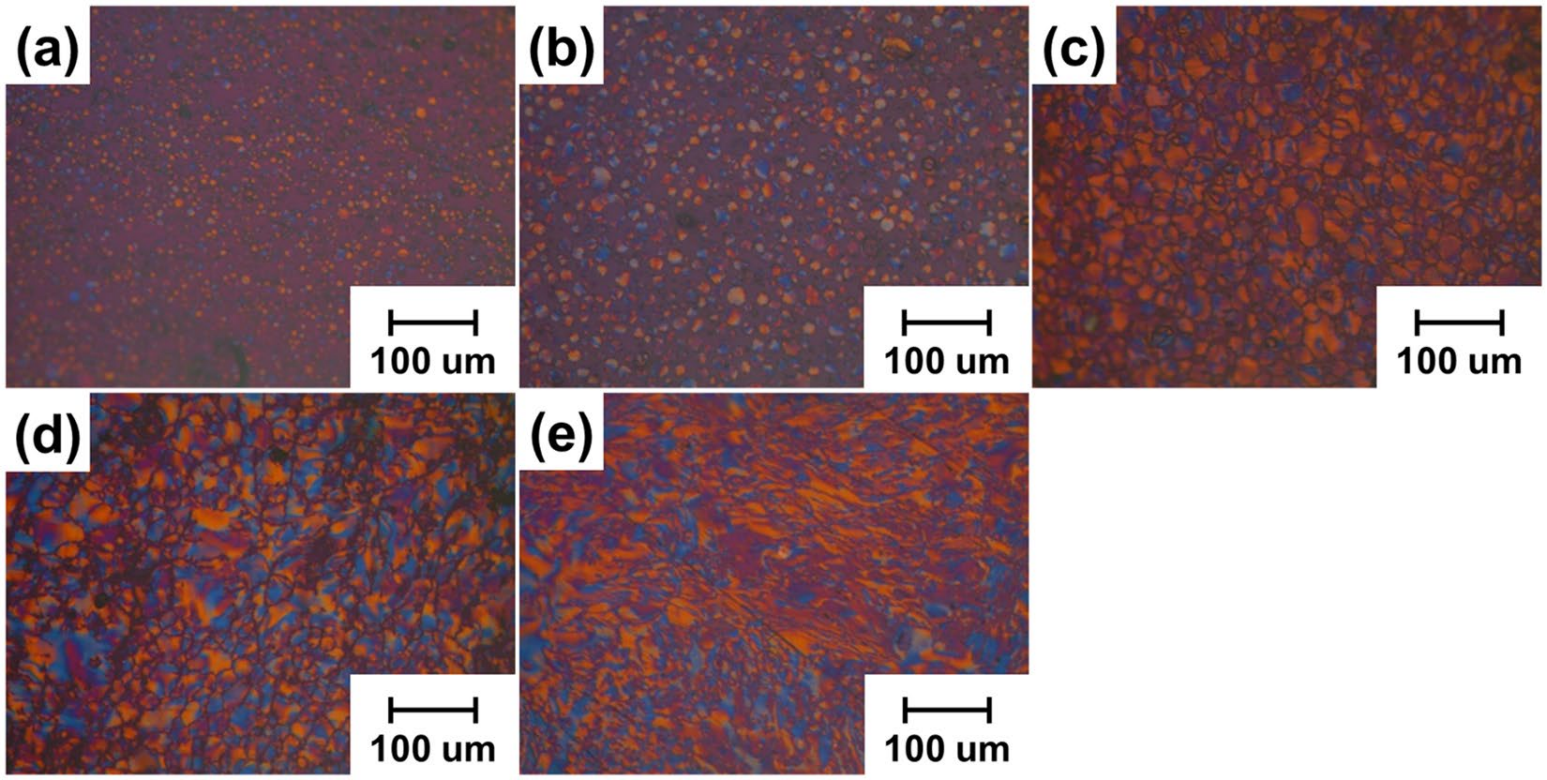
Polarized light microscope analysis of heat treated CTP at different temperature: (**a**) 400 °C; (**b**) 430 °C; (**c**) 450 °C; (**d**) 480 °C; (**d**) 500 °C.

**Figure 2:**
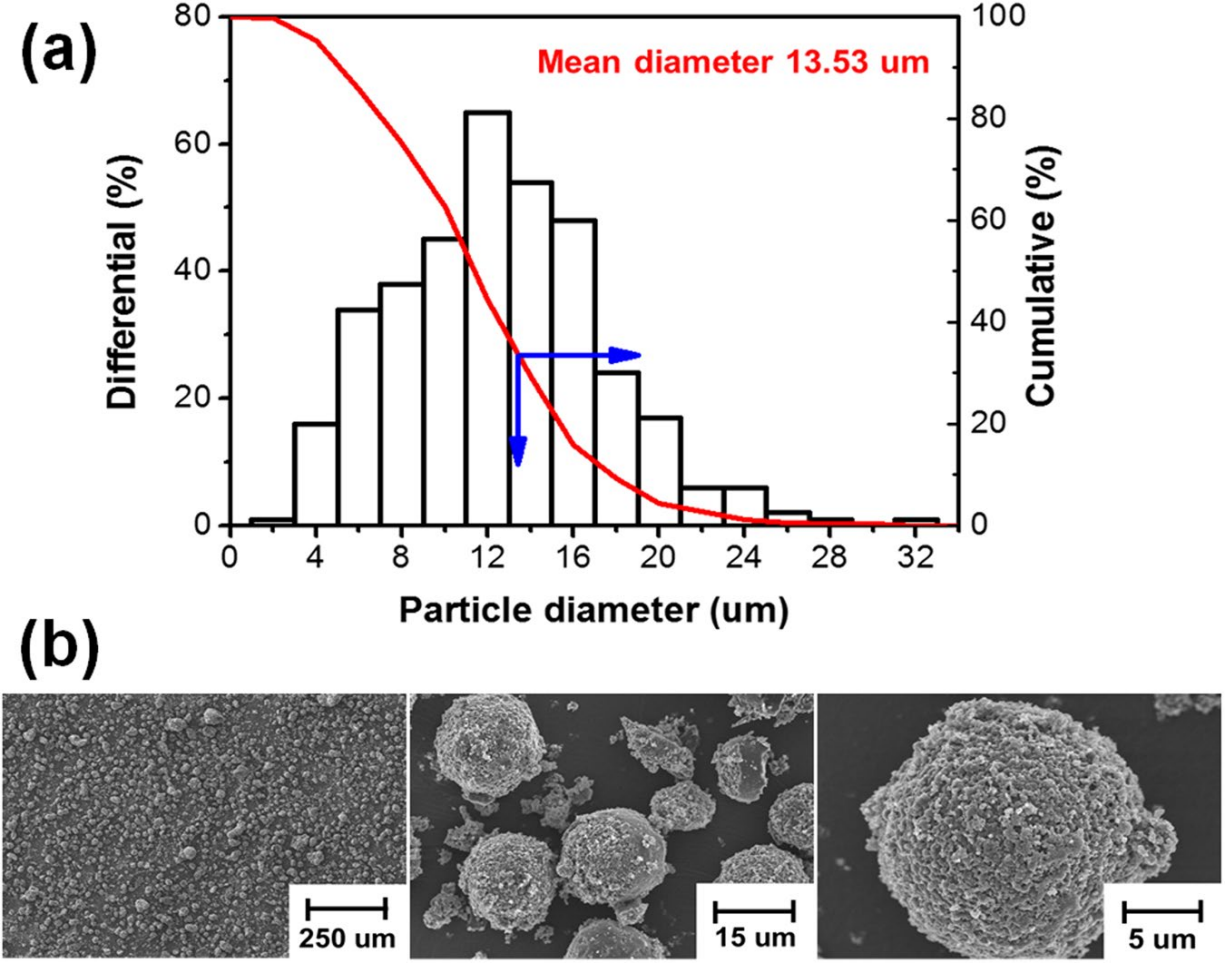
The particle diameter and SEM images of MCMBs produced at 430 °C: (**a**) the particle diameter; (**b**) SEM images.

The CTPs produced at five heat treatment temperatures formed different mesophases, as shown in Fig. 1. The mesophase structure became clear in accordance with the increase of heat treatment temperature. The heat-treated CTP containing mesophase spherules started to appear at 400 °C and the mesophase pitch of complete anisotropic development was exhibited at temperatures above 480 °C. Uniform mesophase spherules less than 75 μm for the production of high density, high strength carbon blocks were observed under heat treatment of 430 °C^5^. Figure 2(a) shows the average particle diameter of the mesophase spherules prepared at 430 °C. All of the mesophase spherules were smaller than 40 μm and the average particle diameter was 13.53 μm. Figure 2(b) shows a SEM image of extracted MCMBs from the CTP shown in Fig. 1(b). Uniformity of around 14 um size was verified via the SEM image, and a rough surface of the MCMBs was confirmed. The yield of MCMBs treated at a growth temperature of 430 °C is over 65% after extraction by tetrahydrofurane (THF).

### MCMB properties with oxidative stabilization

**Figure 3:**
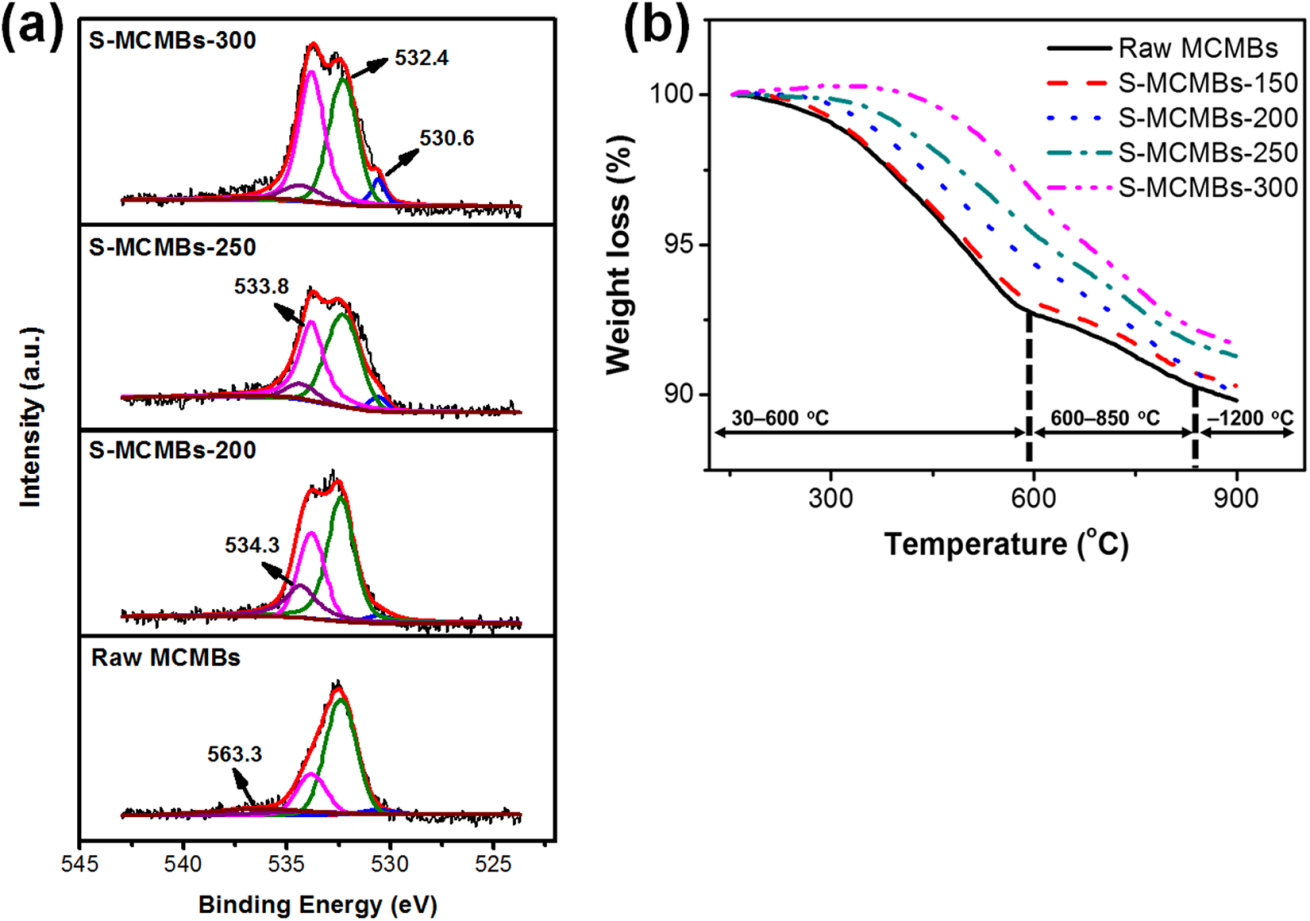
Characteristic analysis of Stabilized MCMBs. (**a**) XPS spectra of Raw MCMBs, S-MCMBs-200, S-MCMBs-250, and S-MCMBs-300. (**b**) Thermogravimetric analysis of Raw and Stabilized MCMBs in nitrogen atmosphere at a heating rate of 5 °C/min.

The results of the elementary analysis of the stabilized and raw MCMBs are summarized in Table 1. As the stabilization temperature was increased, the carbon ratio decreased and the ratio of other components increased. Since a representative element of the other components is the oxygen content, it is considered that the oxygen content also increased as the stabilization temperature was increased. The hydrogen ratio decreased sharply at stabilization temperature above 200 °C. The C/H ratios were 3.69, 2.35, and 2.95 for Raw MCMBs, S-MCMBs-200, and S-MCMBs-300, respectively. From these results, it appears that the aromaticity decreased and increased at the point based on the stabilization temperature of 200 °C. Oxygen content depending on oxidation stabilization temperature determined via X-ray photoelectron spectroscopy (XPS) is shown in Fig. 3(a). The five functional groups analyzed were C=O, C-O, O-C=O, C-OH, and H_2_O (chemisorbed O_2_ or adsorbed H_2_O) at 530.6, 532.4, 533.8, 534.3, and 563.3, respectively^31–33^. With increasing temperature of oxidation stabilization, the functional groups of C=O and O-C=O were increased and H_2_O was removed. S-MCMBs-200 showed more C-OH content than the other samples. Thus, it can be concluded that the hydrogen ratio shown in Table 1 increases or decreases at the stabilization temperature of 200 °C due to the formation of -OH functional groups.

**Table 1:** Elementary analysis of MCMBs according to stabilized condition.

Samples	Elemental analysis [wt%]				Ratio of atoms
	C	H	N	Others	C/H
Raw MCMBs	96.15	2.17	1.32	0.36	3.69
S-MCMBs-150	95.17	3.13	1.05	0.65	2.53
S-MCMBs-200	94.10	3.34	0.78	1.78	2.35
S-MCMBs-250	92.80	2.79	0.84	3.57	2.77
S-MCMBs-300	91.83	2.59	1.14	4.44	2.95

### Mechanical properties of carbonized carbon blocks

Table 2 shows the mechanical properties of carbonized carbon blocks. The mechanical properties of the carbonized carbon block from Raw MCBMs were not analyzed due to the swelling phenomenon. CCB-200 exhibited the highest mechanical properties with a bending strength of 116 Mpa, a volume shrinkage of 31.51%, and a bulk density of 1.57 g/cm^3^. In the previously reported paper^2,4,11,13^, the density of carbonized carbon block from MCMBs without binder was reported to be over 1.7 g/cm^3^, and the flexural strength was noted to be about 90 MPa. Thus, the density of CCB-200 was lower than that of the previous report, but it showed higher flexural strength. In addition, as the content of oxygen increased, the flexural strength was expected to increase continuously, but it tends to decrease when it exceeds a certain amount. From these results, although addition of oxygen has the advantage of restacking the carbon during the carbonization process, it shows that the oxygen content, which involved above a certain amount, has the disadvantage of reducing the flexural strength and decreasing in density.

**Table 2:** Mechanical properties of carbonized carbon blocks from MCMBs.

Sample Name.	Shore hardness [HS]	Bending strength [Mpa]	Volume shrinkage [%]	Bulk density [g/cm^3^]
CCB-150	90	—	20.22	1.35
CCB-200	94	116	31.51	1.57
CCB-250	85	43	29.78	1.48
CCB-300	85	14	29.54	1.42

### Correlation between thermal and morphological properties

Figure 3(b) shows the results of the thermogravimetric analysis of the stabilized MCMBs. There are three ranges based on the slope where the weight loss rate varies. The weight loss rate divided into three ranges from 30 °C to 1200 °C is indicated in Table 3. The fixed carbon of S-MCMBs-150 and S-MCMBs-200 and that of S-MCMBs-250 and S-MCMBs-300 is approximately 0.3% and 1.6% higher than that of Raw MCMBs, respectively. However, the amount of remaining total weight treated in S-MCMBs-250 and S-MCMBs-300 is around 1% less than that of Raw MCMBs, as shown in Table 3. The amount of volatile matter from 30 °C to 600 °C is highest for Raw MCMBs and S-MCMBs-150 whereas S-MCMBs-300 has the largest amount of volatile matter from 850 °C to 1200 °C. From these results, it can be seen that a large amount of oxygen content was released from 850 °C to 1200 °C.

**Table 3:** The amount of change in the weight loss rate of MCMBs classified by three carbonization temperature ranges.

Samples	Weight loss rate [%]			
	Total weight loss	30–600 [°C]	600–850 [°C]	850–1200 [°C]
Raw MCMBs	10.35	7.23	2.47	0.65
S-MCMBs-150	10.31	7.43	2.39	0.49
S-MCMBs-200	10.33	5.93	3.68	0.72
S-MCMBs-250	11.17	5.07	3.65	2.45
S-MCMBs-300	11.76	4.05	4.52	3.20

**Figure 4:**
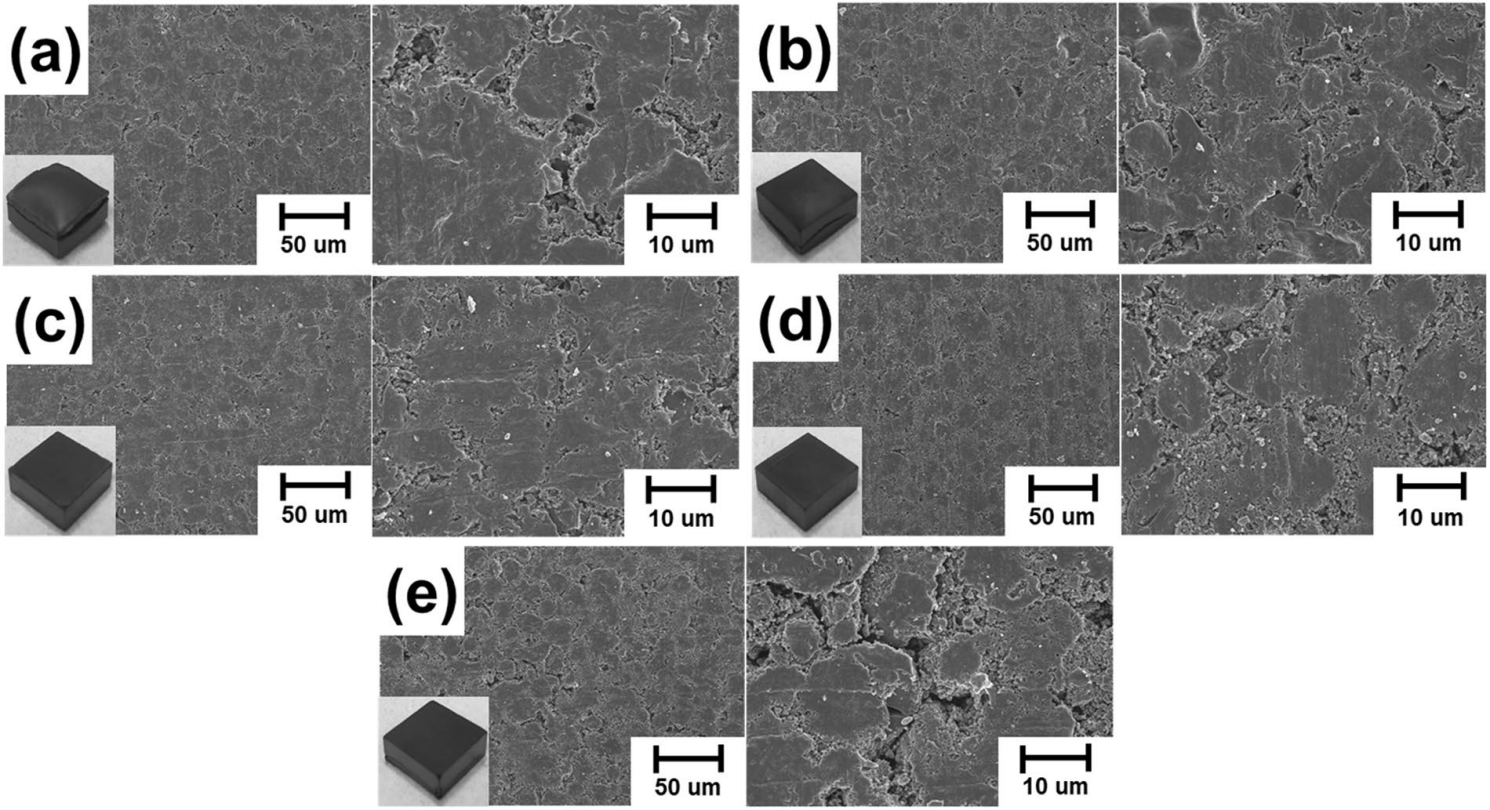
SEM images of CCB treated at different stabilization conditions: (**a**) CCB-Raw MCMBs; (**b**) CCB-150; (**c**) CCB-200; (**d**) CCB-250; (**e**) CCB-300.

Figure 4 shows the morphological analysis of CCB according to the stabilization process conditions. The swelling phenomenon can be observed in Fig. 4(a,b). Hence, the amount of weight loss from 30 °C to 600 °C is considered to be the main factor responsible for the swelling phenomenon. The borderlines among MCMBs particles in Fig. 4(d,e) showing significant weight loss from 850 °C to 1200 °C were distinctly observed^8^. Also, weight loss from 850 °C to 1200 °C was reported as the temperature range as having the smallest volume shrinkage^4^. It is thus concluded that small cracks among MCMBs particles are mainly caused by the amount of weight loss from 850 °C to 1200 °C. Consequently, we verified that oxidative stabilization treated above 250 °C weakens the mechanical properties resulting from the amount of the volatile matter at a certain temperature range from 850 °C to 1200 °C increased by the amount of oxygen contents.

**Figure 5:**
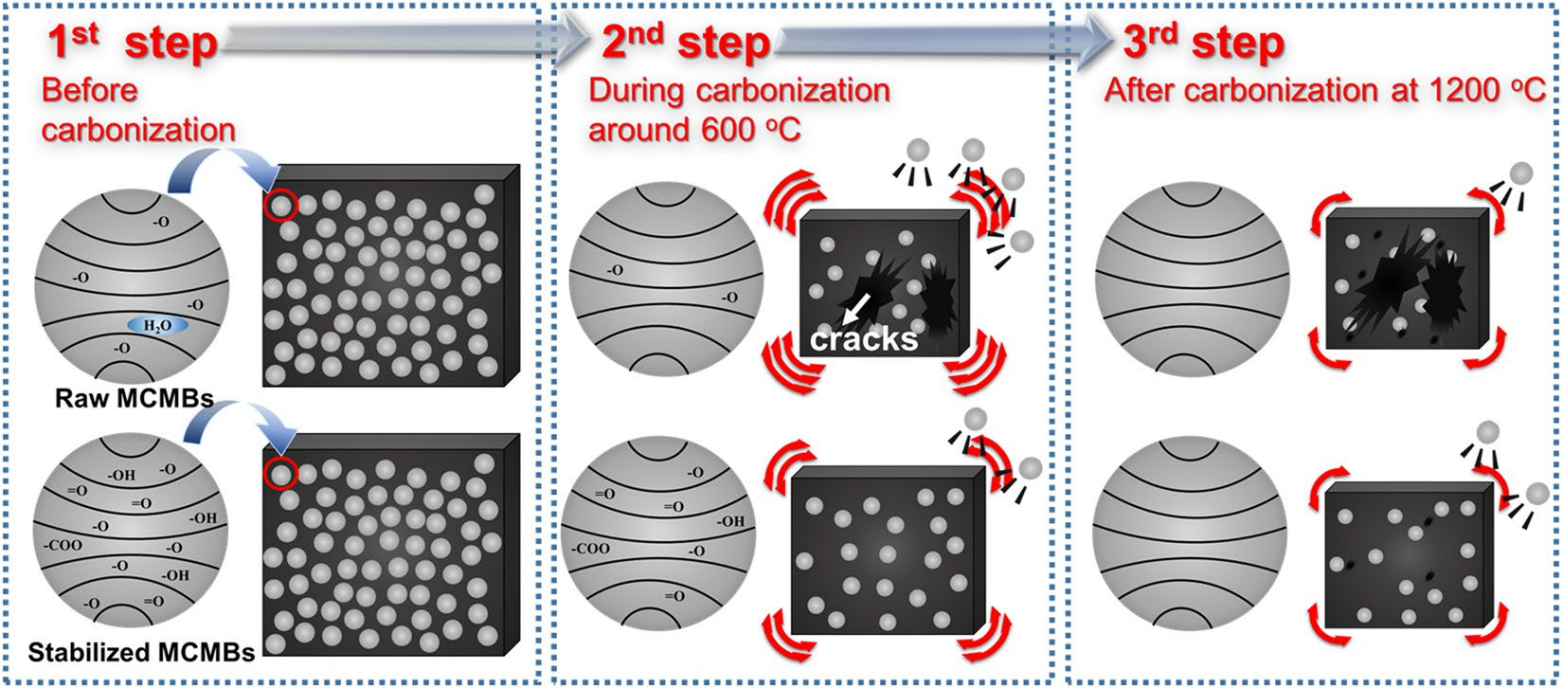
Schematic model of the sintering mechanism of carbon blocks from MCMBs during carbonization.

Based on the above results, it is reasonable to conclude that sintering, as illustrated in Fig. 5, occurs during the carbonization process. The first step shows oxygen content in CCB according to the XPS results. The second step from 30 °C to 600 °C in the carbonization process is explained by abundant volatile matter, considerable volume shrinkage, and the swelling phenomenon of the carbon block manufactured from Raw MCMBs. The final step presents that the weight loss at a temperature range from 850 °C to 1200 °C produced small cracks with a small volume shrinkage.

## Conclusion

From the aforementioned results the following conclusions were drawn. First, the carbon block from Raw MCMBs showed more than 100 um cracks due to the weight loss from 30 °C to 600 °C. Second, as more volatile matter was incorporated corresponding to the weight loss from 850 °C to 1200 °C, more borderlines among MCMBs particles distinctly appeared. In addition, the bending strength was decreased. Third, carbon blocks from stabilized MCMBs have higher volume shrinkage and weight loss rate than carbon blocks from Raw MCMBs. Fourth, bending strength decreased as the oxygen content increased at stabilization temperature over 250 °C. Finally, in order to manufacture high density carbon blocks that are carbonized at 1200 °C, the MCMBs should be stabilized at temperature in a temperature range of 150–250 °C under an air atmosphere.

## Methods

### Materials

**Figure 6:**
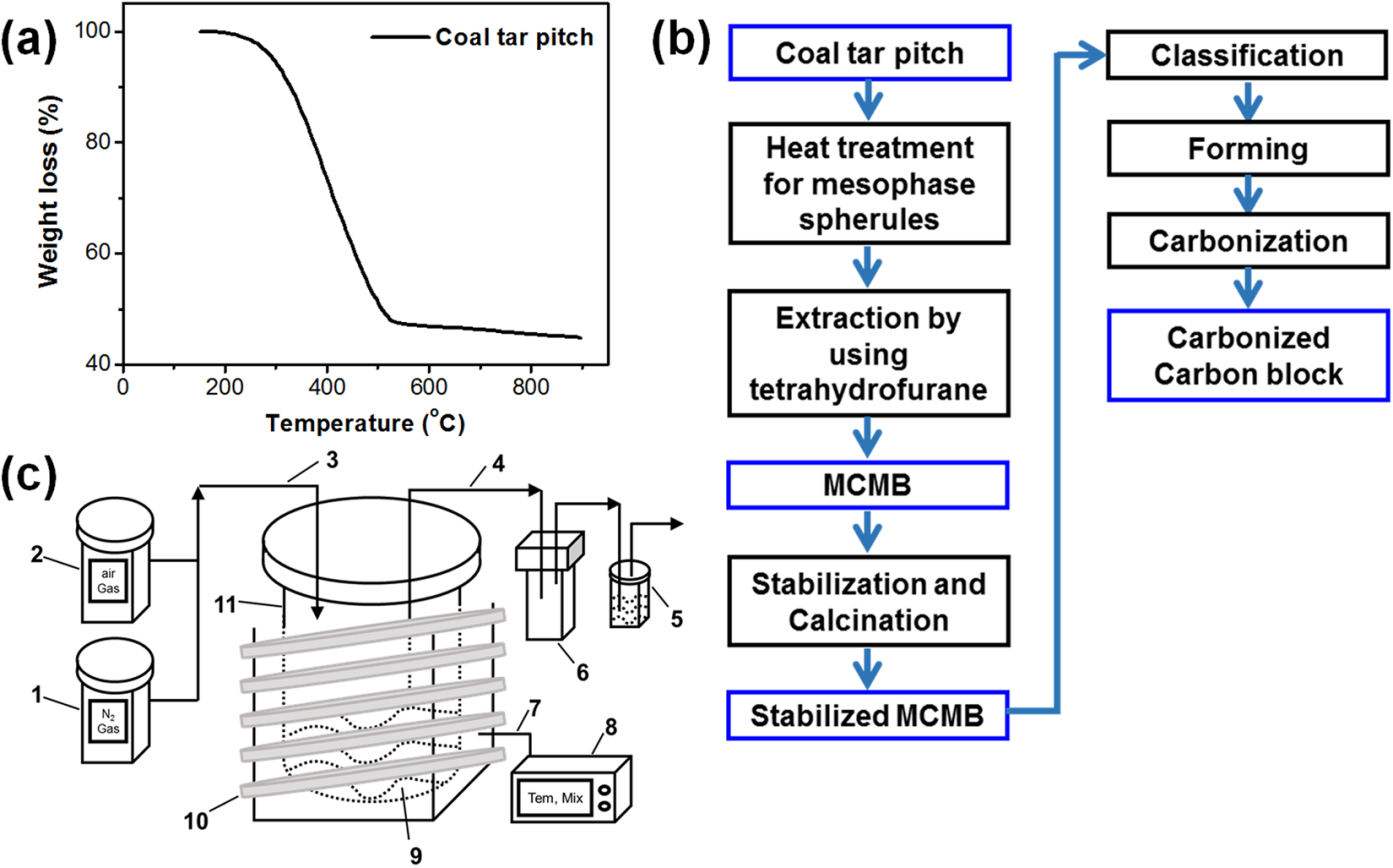
Raw materials properties and experimental design. (**a**) TG weight loss curve of CTP. (**b**) Experimental flow chart for carbon block produced from MCMBs. (**c**) Apparatus for heat treatment and oxidative stabilization, (1) air gas system, (2) nitrogen gas system, (3) gas inlet line, (4) gas outlet line, (5) water trap, (6) stores for distillates, (7) thermocouple, (8) temperature controller, (9) coal tar pitch, (10) heating coils, (11) reactor.

The raw material used in this study was coal tar pitch (CTP) obtained from OCI Company Ltd in Korea. The characteristics of the coal tar pitch are listed in Table 4. An elemental analysis (EA) of the coal tar pitch revealed the following: carbon (93.50%), hydrogen (4.49%), nitrogen (1.34%), and sulfur (0.46%). A solubility analysis based on Quinoline Insolubility (QI) and Toluene Insolubility (TI) was also carried out. The carbon yield of CTP was 44.9% at 900 °C, as indicated in Fig. 6(a).

**Table 4:** Characteristics of the coal tar pitch.

Elemental analysis [wt %]				C/H	Insolubility analysis [%]	
C	H	N	S		TI	QI
93.5	4.49	1.34	0.46	1.74	32.1	9.7

### Preparation of MCMBs

Figure 6(b) shows the experimental procedure to manufacture carbonized carbon blocks from MCMBs. CTP, employed as raw material, was heated at 400–500 °C under a nitrogen atmosphere by using the reactor presented in Fig. 6(c). Heated coal tar pitch in a quantity of 10 g was mixed with tetrahydrofurane (THF) of 100 ml at 50 °C for 12 h, and then extracted by vacuum filtration. Additionally, the extracted MCMBs were washed twice with toluene at 80 °C. The as-prepared MCMBs were heated under an air atmosphere at 150–300 °C for 1 h to increase oxygen content, and this heat treatment was carried out in the device shown in Fig. 6(c). Four kinds of MCMBs according to the stabilization process were manufactured at 150, 200, 250, 300 °C, and respectively designated as S-MCMBs-150, S-MCMBs-200, S-MCMBs-250, and S-MCMBs-300.

### Manufacturing carbon blocks

First, raw MCMBs and stabilized MCMBs were classified in a sieve under 75 um (200 mesh). The raw MCMBs and stabilized MCMBs were then molded without a binder under 28 Mpa by cold compression into two discs of 15 × 15 × 3 mm and 60 × 10 × 3 mm^5^. The green carbon blocks were carbonized at 1200 °C for 1 h with a heating rate of 1 °C/min in a nitrogen atmosphere. Two types of carbonized carbon blocks (CCB) from MCMBs were then prepared. Also, four kinds of CCB which treated by different stabilization temperatures respectively at 150, 200, 250, 300 °C, were classified as CCB-150, CCB-200, CCB-250, and CCB-300.

### Characterization

The composition of CTP and MCMBs was analyzed using an elemental analysis (EA, TruSpec, LECO Corp., USA). Toluene insolubility (TI) and Quinoline insolubility (QI) were respectively determined according to ASTM D 4072 and ASTM D 2318 standards. The thermal behavior of the CTP and MCMBs was analyzed by a thermogravimetric analysis (TGA, STA 409 PC, Netzsch Corp., Germany) at a heating rate of 5 °C/min to 900 °C in a nitrogen flow. A polarization microscopy analysis and a particle diameter analysis were carried out using polarized light microscopy (PLM, BX51M, Olympus Corp., Japan). The surface images of the MCMBs and CCBs was obtained using scanning electron microscopy (SEM, JSM-6700F, JEOL Ltd., Japan). The oxygen content of MCMBs was determined via X-ray photoelectron spectroscopy (XPS, AXIS-NOVA, Kratos Inc., Japan). The density of the CCBs was investigated by the Archimedes drainage method. The shore hardness test (SH, Type-D, Kobunshi Keiki, Japan) was performed in accordance with the ASTM D 2240 standard. The flexural strengths were examined by a Universal Testing Machine (UTM, WL2100, WITHLAB Ltd., Korea) according to equation (1)^13,34,35^:

1 $${\sigma }_{f}=\frac{3\cdot P\cdot L}{2\cdot w\cdot {t}^{2}}$$

where *P* is the breaking force of the specimen, and *L* is the span of bending test (30 mm), *w* is the width of specimen (10 mm), *t* is the thickness of the specimen (3 mm).

## Electronic supplementary material

Figure Caption

Table Caption

## References

[1] Miyazaki K, Hagio T, Kobayashi K (1981). Graphite and boron carbide composites made by hot-pressing. Journal of Materials Science.

[2] Delport MR, Badenhorst H (2016). Production of a self-adhering mesophase powder from anthracene oil for low pressure forming of graphite artefacts. Journal of Materials Science.

[3] Shen K (2015). Advantages of natural microcrystalline graphite filler over petroleum coke in isotropic graphite preparation. Carbon.

[4] Wang Y-G, Korai Y, Mochida I (1999). Carbon disc of high density and strength prepared from synthetic pitch-derived mesocarbon microbeads. Carbon.

[5] Lee S-M, Kang D-S, Roh J-S (2015). Bulk graphite: materials and manufacturing process. Carbon letters.

[6] Lin Q, Li T, Zheng C, Zhao Y, Song S (2004). Carbonization behavior of coal-tar pitch modified with divinylbenzene and optical texture of resultant semi-cokes. Journal of Analytical and Applied Pyrolysis.

[7] Fang M-D (2012). Improving the self-sintering of mesocarbon-microbeads for the manufacture of high performance graphite-parts. Carbon.

[8] Shen K, Huang Z-H, Yang J, Shen W, Kang F (2011). Effect of oxidative stabilization on the sintering of mesocarbon microbeads and a study of their carbonization. Carbon.

[9] Yamada Y (1974). Characteristics of meso-carbon microbeads separated from pitch. Carbon.

[10] Manocha LM, Patel M, Manocha SM, Vix-Guterl C, Ehrburger P (2001). Carbon/carbon composites with heat-treated pitches. Carbon.

[11] Fang M-D (2015). Improving the sintering behavior of mesocarbon-microbeads for the manufacture of high quality carbon products using a joint promoter comprising carbon black and glycidyl methacrylate. Materials Chemistry and Physics.

[12] Cheng Y (2013). *In situ* preparation and mechanical properties of CNTs/MCMBs composites. Composites Part B: Engineering.

[13] Song Y (2004). Carbon/graphite seal materials prepared from mesocarbon microbeads. Carbon.

[14] Broussely M, Biensan P, Simon B (1999). Lithium insertion into host materials: the key to success for Li ion batteries. Electrochimica Acta.

[15] Endo M, Kim C, Nishimura K, Fujino T, Miyashita K (2000). Recent development of carbon materials for Li ion batteries. Carbon.

[16] Mochida I, Korai Y, Ku C-H, Watanabe F, Sakai Y (2000). Chemistry of synthesis, structure, preparation and application of aromatic-derived mesophase pitch. Carbon.

[17] Lei, Y. *et al*. Porous mesocarbon microbeads with graphitic shells: constructing a high-rate, high-capacity cathode for hybrid supercapacitor. *Scientific Reports* **3**, 2477, 10.1038/srep02477, https://www.nature.com/articles/srep02477#supplementary-information (2013). 10.1038/srep02477 PMC3748843 23963328

[18] Drbohlav J, Stevenson WTK (1995). The oxidative stabilization and carbonization of a synthetic mesophase pitch, part I: The oxidative stabilization process. Carbon.

[19] Zeng SM, Maeda T, Tokumitsu K, Mondori J, Mochida I (1993). Preparation of isotropic pitch precursors for general purpose carbon fibers (GPCF) by air blowing—II. Air blowing of coal tar, hydrogenated coal tar, and petroleum pitches. Carbon.

[20] Mochida I (1990). Preparation of mesophase pitch from aromatic hydrocarbons by the aid of HFBF3. Carbon.

[21] Korai Y, Nakamura M, Mochida I, Sakai Y, Fujiyama S (1991). Mesophase pitches prepared from methylnaphthalene by the aid of HFBF3. Carbon.

[22] Bismarck A (1999). Study on surface and mechanical fiber characteristics and their effect on the adhesion properties to a polycarbonate matrix tuned by anodic carbon fiber oxidation. Composites Part A: Applied Science and Manufacturing.

[23] Jones LE, Thrower PA (1991). Influence of boron on carbon fiber microstructure, physical properties, and oxidation behavior. Carbon.

[24] Mimeault VJ (1971). Carbon fiber composites: Effect of fiber oxidation on composite behavior. Fibre Science and Technology.

[25] Hummers WS, Offeman RE (1958). Preparation of Graphitic Oxide. Journal of the American Chemical Society.

[26] Chen J, Yao B, Li C, Shi G (2013). An improved Hummers method for eco-friendly synthesis of graphene oxide. Carbon.

[27] Shen J (2009). Fast and Facile Preparation of Graphene Oxide and Reduced Graphene Oxide Nanoplatelets. Chemistry of Materials.

[28] Bai H, Li C, Shi G (2011). Functional Composite Materials Based on Chemically Converted Graphene. Advanced Materials.

[29] Wang G (2012). Flexible Pillared Graphene-Paper Electrodes for High-Performance Electrochemical Supercapacitors. Small.

[30] Su C-Y (2010). Highly Efficient Restoration of Graphitic Structure in Graphene Oxide Using Alcohol Vapors. ACS Nano.

[31] Arrigo R (2010). Tuning the Acid/Base Properties of Nanocarbons by Functionalization via Amination. Journal of the American Chemical Society.

[32] Jin Z, Yan X, Yu Y, Zhao G (2014). Sustainable activated carbon fibers from liquefied wood with controllable porosity for high-performance supercapacitors. Journal of Materials Chemistry A.

[33] Li Z, Wu L, Liu H, Lan H, Qu J (2013). Improvement of aqueous mercury adsorption on activated coke by thiol-functionalization. Chemical Engineering Journal.

[34] Song Y-z (2006). Seal and wear properties of graphite from MCMBs/pitch-based carbon/phenolic-based carbon composites. Carbon.

[35] Patton RD, Pittman JCU, Wang L, Hill JR (1999). Vapor grown carbon fiber composites with epoxy and poly(phenylene sulfide) matrices. Composites Part A: Applied Science and Manufacturing.

