# Supplementary material for: Improving the mechanical properties of a high density carbon block from mesocarbon microbeads according to oxidative stabilization
Source: Sci Rep. 2018 Jul 23;8:11064. doi: 10.1038/s41598-018-26971-8 (PMC6056457; doi:10.1038/s41598-018-26971-8)
Supplement: Supplementary file 1 — Figure Caption [file 41598_2018_26971_MOESM1_ESM.docx]

**Figure Captions**

**Figure 1.** Polarized light microscope analysis of heat treated CTP at different temperature: (a) 400 ^o^C; (b) 430 ^o^C; (c) 450 ^o^C; (d) 480 ^o^C; (d) 500 ^o^C

**Figure 2.** The particle diameter and SEM images of MCMBs produced at 430 ^o^C: (a) the particle diameter; (b) SEM images.

**Figure 3.** Characteristic analysis of Stabilized MCMBs: (a) XPS spectra of Raw MCMBs, S-MCMBs-200, S-MCMBs-250, and S-MCMBs-300; (b) Thermogravimetric analysis of Raw and Stabilized MCMBs in nitrogen atmosphere at a heating rate of 5 ^o^C/min.

**Figure 4.** SEM images of CCB treated at different stabilization conditions: (a) CCB-Raw MCMBs; (b) CCB-150; (c) CCB-200; (d) CCB-250; (e) CCB-300.

**Figure 5.** Schematic model of the sintering mechanism of carbon blocks from MCMBs during carbonization.

**Figure 6.** Raw materials properties and experimental design: (a) TG weight loss curve of CTP; (b) Experimental flow chart for carbon block produced from MCMBs; (c) Apparatus for heat treatment and oxidative stabilization, (1) air gas system, (2) nitrogen gas system, (3) gas inlet line, (4) gas outlet line, (5) water trap, (6) stores for distillates, (7) thermocouple, (8) temperature controller, (9) coal tar pitch, (10) heating coils, (11) reactor.
