# Supplementary material for: Improving the mechanical properties of a high density carbon block from mesocarbon microbeads according to oxidative stabilization
Source: Sci Rep. 2018 Jul 23;8:11064. doi: 10.1038/s41598-018-26971-8 (PMC6056457; doi:10.1038/s41598-018-26971-8)
Supplement: Supplementary file 2 — Table Caption [file 41598_2018_26971_MOESM2_ESM.docx]

**Table Captions**

**Table 1**. Elementary analysis of MCMBs according to stabilized condition.

**Table 2.** Mechanical properties of carbonized carbon blocks from MCMBs.

**Table 3.** The amount of change in the weight loss rate of MCMBs classified by three carbonization temperature ranges.

**Table 4.** Characteristics of the coal tar pitch.
